# Editorial: Muscular dystrophies: Current therapeutic advances to improve and restore muscle homeostasis

**DOI:** 10.3389/fcell.2022.1009439

**Published:** 2022-08-31

**Authors:** Ariadna Bargiela, Francisco Hernández-Torres

**Affiliations:** ^1^ Translational Genomics Group, University Institute for Biotechnology and Biomedicine (BIOTECMED), University of Valencia, Valencia, Spain; ^2^ Cardiac and Skeletal Myogenesis Group, MEDINA Foundation, Center for Excellence in Research of Innovative Medicines in Andalusia, Granada, Spain; ^3^ Department of Biochemistry, Molecular Biology III and Immunology, School of Medicine, University of Granada, Granada, Spain

**Keywords:** muscular stem cells, muscular dystrophies, regeneration, therapy, muscle differentiation

Skeletal muscle is a heterogeneous tissue representing between 30 and 38% of the human body mass and has important functions in the organism, such as maintaining posture, locomotor impulse, or pulmonary ventilation (Janssen et al., 2000). Many disorders can affect this tissue. Among them, Muscular Dystrophies (MDs) constitute the most important group in terms of the number of people affected and economic impact generated in public health systems (Emery, 1991; Cavazza et al., 2016; Flores et al., 2020). These pathologies are inherited myogenic disorders characterized by progressive muscle wasting and weakness of variable distribution and severity, where muscle maintenance is compromised. By editing the Research Topic: *“Muscular Dystrophies: Current Therapeutic Advances to Improve and Restore Muscle Homeostasis”* we have attempted to compile some of the latest advances in the field of DMs research.

Six primary research manuscripts were chosen to be part of this Research Topic. Marini et al. generated cardiac organoids from Duchenne Muscular Dystrophy (DMD) patients-derived pluripotent stem cells and studied if DMD-related cardiomyopathy and disease progression occur in these organoids upon long-term culture. Interestingly, these patient-derived cardiac organoids displayed DMD-related cardiomyopathy and disease progression phenotypes. Certainly, this 3D human cardiac model constitutes a valuable tool to study DMD-related cardiomyopathies *in vitro*. Also in DMD, Farea et al. characterize Dp71 ab an isoform of the dystrophin protein which lacks exon 71 and 78 and compares to Dp71. Authors demonstrate that Dp71 ab promotes myoblasts proliferation while Dp71 has an opposite effect. By means of reporter constructs authors studied subcellular localization of both isoforms and concluded that Dp71 ab is a myoblast-specific proliferation enhancer as no activity was observed in non-myoblasts cells. In the field of limb girdle muscular dystrophies, Lasa-Elgarresta et al. investigate the potential contribution of the ubiquitin-proteasome system (UPS) to increased sarco/endoplasmic reticulum calcium ATPase (SERCA) degradation in Limb-girdle muscular dystrophy recessive 1 (LGMDR1). The authors demonstrate reduced SERCA expression in several cellular and animal models of LGMDR1, an early preclinical feature in CAPN3-deficient mice. These results support the involvement of SERCA in the etiopathology of LGMDR1 and lay the foundation to explore SERCA2 as well as UPS as novel molecular targets for the treatment of LGMDR1 muscular dystrophy. In the fourth highlighted manuscript, Diez-Fuertes et al. unravel how immune response activation in individuals with Limb Girdle Muscular Dystrophy Dominant 2 (LGMDD2) contributes to resistance to HIV-1 infection. The analysis performed in this study suggests a pro-inflammatory state in LGMDD2 patients, also described for other muscular dystrophies, that is characterized by the alteration of IL-17 signaling pathway and the consequent increase of metallopeptidases activity and TNF response. Thus, the increase in interferons and inflammatory mediators seems to generate an antiviral environment and resistance to HIV-1 infection but that could also impair muscular function in LGMDD2 patients. Overall, the investigation of the main pathways found altered and associated with LGMDD2 pathology has obvious implications in the design of new therapeutic strategies, not only for this type of muscular dystrophy but also for HIV-1 infection. DUX4 is a transcription factor and its missregulation is linked to Facioscapulohumeral muscular dystrophy (FSHD). DUX4 binds DNA by two homodomains identical to those found in DUX4c, another transcriptional regulator. However, very little is known about the involvement of DUX4c in FSHD. To shed light to this fact Ganassi et al. investigated DUX4c interferes with DUX4 function in human myogenesis. Authors found an antagonistic interplay between both members as DUX4 alters cell viability via β-CATENIN signalling and DUX4c counteracts aspects of DUX4-mediated toxicity in human muscle cells, potentially acting as a gene modifier for severity. Late-onset Pompe disease (LOPD) is an autosomal-recessive metabolic myopathy with an available treatment based on enzyme replacement since 2006. Ravaglia et al. evaluate the response to the therapy of 15 patients followed up for 15 years. Authors conclude that Body Impedance Vectorial Analysis for serial body composition assessment is a valuable and easy-employable tool to predict response of LOPD patients to long-term treatments. Additionally, they conclude that Low Phase Angle could quantitatively measure muscle quality, useful for predict response of a patient to a treatment such enzyme replacement. Thus, those patients with poorest predictions can be identified and get a closer medical control.

Overall, considering that muscular dystrophies represent a group of disorders that directly affect muscular tissue and because of their high incidence worldwide, the editors were looking for scientific advance in the knowledge of this group of pathologies. This topic makes available to the scientific community a compendium of publications of high scientific level related to the subject including publications on Duchenne, Pompe or limb-girdle muscular dystrophies.
